# Relationship between parity and the problems that appear in the postpartum period

**DOI:** 10.1038/s41598-019-47881-3

**Published:** 2019-08-13

**Authors:** Juan Miguel Martínez-Galiano, Antonio Hernández-Martínez, Julián Rodríguez-Almagro, Miguel Delgado-Rodríguez, Juan Gómez-Salgado

**Affiliations:** 1 Department of Nursing of University of Jaen, Jaen, Spain; 2 CIBER de Epidemiología y Salud Pública (CIBERESP), Madrid, Spain; 3 Mancha-Centro Hospital, Alcázar de San Juan (Ciudad Real), Ciudad Real, Spain; 4 Department of Nursing, University of Castilla la Mancha, Ciudad Real, Spain; 5 Department of Health Sciences of University of Jaen, Jaen, Spain; 6 Department of Sociology, Social Work and Public Health, University of Huelva, Huelva, Spain; 7 Safety and Health Postgrade Program, Universidad Espíritu Santo, Guayalquil, Ecuador

**Keywords:** Quality of life, Outcomes research

## Abstract

Parity is associated with the incidence of problems in pregnancy, delivery and the puerperium. The influence of parity in the postpartum period has been poorly studied and the results are incongruous. The objective of this study was to identify the association between parity and the existence of distinct discomfort and problems during the postpartum period. Cross-sectional study with puerperal women in Spain. Data was collected on demographic and obstetric variables and maternal manifestations of discomfort and problems during the postpartum period. An ad hoc online questionnaire was used. Crude odds ratios (ORs) and adjusted odds ratios (aORs) with 95% confidence intervals (CIs) were estimated by conditional logistic regression. 1503 primiparous and 1487 multiparous participated in the study. 53.4% (803) of the primiparous women affirmed to have feelings of sadness, as opposed to 36.2% (539) of multiparous women (aOR: 1.60; 95% CI: 1.35–1.89). 48.3% (726) of primiparous had lactation problems vs 24.7% (367) of multiparous (aOR: 2.46; 95% CI: 2.05–2.94). 37.2% (559) of primiparous reported anxiety, while the percentage in multiparous was 25.7% (382) (aOR: 1.34; 95% CI: 1.12–1.61). 22.2% (333) of primiparous had depressive symptoms, and 11.6% (172) of multiparous (aOR: 1.65; CI 95%: 1.31–2.06). Faecal incontinence was more present in primiparous than in multiparous, 6.5% (97) and 3.3% (49) respectively (aOR: 1.60; 95% CI: 1.07–2.38). Parity is associated with the presence of certain problems in the postpartum period. Thus, primiparous are more likely to have lactation problems, depressive symptoms, anxiety, sadness, and faecal incontinence.

## Introduction

Parity is a term that defines the number of children that a woman has. We need to differentiate between the number of pregnancies and the number of children and births, since there may have been a pregnancy that did not complete itself with a birth. In obstetrics, in general, and in assisting the process of pregnancy, childbirth and, particularly, the puerperium period, the parity parameter is taken into account in the daily clinical practice^1^, although this concept is not equally understood by all professionals involved in perinatal care^2^.

The effect of parity on different health problems such as cancer, bone fractures, biliary lithiasis, diabetes and uterine prolapse events has been studied^3–7^. Also, the role that parity has in several parameters and pathologies during the pregnancy, childbirth and puerperium processes has undergone numerous investigations^8–13^. A cross-sectional study carried out in Peru on 222 women identified an association between women’s parity and the incidence of depression during pregnancy^8^. No association was found in the abandonment of exclusive lactation and parity in a Venezuelan study on 106 women^9^. The results of a study with 380 women in Denmark suggest that parity should be taken into account to choose the dose of insulin in pregnant women with diabetes type 1^12^. A study carried out in the South of Spain for 6 years detected that parity was related with the performance of episiotomy during childbirth, being primiparity a risk factor^13^.

Different investigations have studied the relation between parity and pregnancy, childbirth and puerperium problems^8–13^. However, few have focused on studying the association between parity and the problems that appear in the postpartum period in a specific way^9^. In fact, most studies that relate parity with the process of pregnancy, childbirth and the puerperium focus on the delivery and, to a lesser extent, the pregnancy^8,10–13^. The scarce results on the subject show inconsistent outcomes and encourage new research. The objective to determinate of the association between parity and the discomfort and problems women present in the postpartum period was proposed.

## Results

1503 primiparous women (50.3%) and 1487 multiparous women (49.7%) participated in the study. In the primiparous women, 44.3% (666) were 35 years old or more, compared to 58.8% (875) of multiparous women. An age ≥35 years was negatively associated with parity (OR: 0.56; CI 95%: 0.48–0.64). In the primiparous group, 96.1% (n = 1445) of cases had middle or high level studies vs. 93.6% (1392) of the multiparous group (OR: 1.70; CI 95%: 1.22–2.38). The attendance to childbirth classes, as part of the health education programme was of 76.7% (1153) in primiparous women and of 42.8% (637) in multiparous (OR: 4.40; CI 95%: 3.76–5.15). As for the birth type, 24.8% (372) of the primiparous group ended in instrumental delivery and 30.5% (459) in caesarean section, while among the multiparous group, the rate was 11.2% (167) and 18.4% (274) respectively. In primiparous women, there was a higher number of deliveries that ended in a distocic form (instrumental and cesarean sections) compared to multiparous group, in the instrumental delivery OR was 3.47 (CI 95%: 2.82–4.26) and in the caesarean section OR was 2.61 (CI 95%: 2.18–3.18).

Furthermore, 36.7% (552) of primiparous mothers’ children received artificial feeding at hospital discharge, while for multiparous mothers’ children, this percentage was 21.9% (326) (OR: 2.07; CI 95%: 1.76–2.43). In Table 1, the other characteristics of the study sample and the factors associated with parity can be seen.

**Table 1:** Characteristics of the studied women associated to parity.

Variable	Total, n	Primiparous N = 1503	Multiparous N = 1487	OR CI 95%
		n (%)	n (%)	
Maternal age				
<35 years	1449	837 (55.7)	612 (41.2)	1 (ref.)
>=35 years	1541	666 (44.3)	875 (58.8)	**0**.**56** (**0**.**48**–**0**.**64**)
Academic level				
No studies/Primary	154	58 (3.9)	95 (6.4)	1 (ref.)
Secondary/University	2837	1445 (96.1)	1392 (93.6)	**1**.**70** (**1**.**22**–**2**.**38**)
Spanish nationality				
No	2886	1445 (96.1)	1441 (96.9)	1 (ref.)
Yes	104	58 (3.9)	46 (3.1)	1.26 (0.85–1.86)
Twin pregnancy				
No	2870	1424 (94.7)	1446 (97.2)	1 (ref.)
Yes	120	79 (5.3)	41 (2.8)	**1**.**96** (**1**.**33**–**2**.**87**)
Gestational age				
On term	2227	1377 (91.6)	1380 (92.8)	1 (ref.)
Preterm	233	126 (8.4)	107 (7.2)	1.18 (0.90–1.54)
Attending childbirth classes				
No	1200	350 (23.3)	850 (57.2)	1 (ref.)
Yes	1790	1153 (76.7)	637 (42.8)	**4**.**40** (**3**.**76**–**5**.**15**)
Problems during pregnancy				
No	2590	1314 (87.4)	1276 (85.8)	1 (ref.)
Yes	400	189 (12.6)	211 (14.2)	0.87 (0.70–1.07)
Induced labour				
No	1992	917 (61.0)	1075 (72.3)	1 (ref.)
Yes	998	586 (39.0)	412 (27.7)	**1**.**67** (**1**.**43**–**1**.**95**)
Epidural use/Spinal anaesthesia				
No	672	214 (14.2)	458 (30.8)	1 (ref.)
Yes	2318	1289 (85.8)	1029 (69.2)	**2**.**68** (**2**.**24**–**3**.**22**)
Type of delivery				
Normal	1718	672 (44.7)	1046 (70.3)	1 (ref.)
Instrumental	539	372 (24.8)	167 (11.2)	**3**.**47** (**2**.**82**–**4**.**26**)
Caesarean	733	459 (30.5)	274 (18.4)	**2**.**61** (**2**.**18**–**3**.**12**)
Perineal tear (III/IV degree)				
No	2865	1425 (94.8)	1440 (96.8)	1 (ref.)
Yes	125	78 (5.2)	47 (3.2)	**1**.**68** (**1**.**16**–**2**.**43**)
Episiotomy				
No	1901	881 (58.6)	1020 (68.6)	1 (ref.)
Yes	1089	622 (41.4)	467 (31.4)	**1**.**54** (**1**.**33**–**1**.**79**)
Skin-to-skin				
No	969	576 (38.3)	393 (26.4)	1 (ref.)
Yes	2021	927 (61.7)	1094 (73.6)	**0**.**58** (**0**.**50**–**0**.**68**)
Newborn admittance				
No	2741	1356 (90.2)	1385 (93.1)	1 (ref.)
Yes	249	147 (9.8)	102 (6.9)	**1**.**47** (**1**.**13**–**1**.**92**)
Artificial feeding at discharge				
No	2112	951 (63.3)	1161 (78.1)	1 (ref.)
Yes	878	552 (36.7)	326 (21.9)	**2**.**07** (**1**.**76**–**2**.**43**)

Table 2 shows the association between different puerperal discomforts and complications and parity. The reference category was multiparity, so the calculated Odds Ratios represent the probability of primiparous of suffering problems/discomfort as compared to multiparous. In this table, 43.6% (656) of primiparous women presented haemorrhoids versus 49.2% (731) of multiparous women (aOR: 0.80; CI 95%: 0.68–0.94). Headache were present in 21.2% (319) of primiparous, while this percentage was 24.1% (358) in multiparous (aOR: 0.75; CI 95%: 0.61–0.91). On the other hand, 46.7% (702) of primiparous women reported sexual problems in the postpartum period, while 31.8% (473) of multiparous reported having this type of problems (aOR: 1.46; CI 95%: 1.23–1.73). Also, couple problems were more frequent in primiparous than in multiparous 34.8% (523) vs 25.2% (375) (aOR: 1.43; CI 95%: 1.20–1.71). In addition, the results show that primiparous presented a greater frequency of lactation problems (aOR: 2.46; CI 95%: 2.05–2.94), depressive symptoms (aOR: 1.65; CI 95%: 1.31–2.06), feelings of sadness (aOR: 1.60; CI 95%: 1.35–1.89), feelings of anxiety (aOR: 1.34; CI 95%: 1.12–1.99) and faecal incontinence (aOR: 1.60; CI 95%: 1.07–2.38) than multiparous women, as can be seen in Table 2.

**Table 2:** Association between parity and problems/discomfort after 6 postpartum weeks.

Variable	Total, n	Primiparous n (%)	Multiparous n (%)
**Constipation** ^**a**^			
No	1743	879 (58.5)	864 (58.1)
Yes	1247	624 (41.5)	623 (41.9)
cOR CI 95%		0.99 (0.85–1.14)	1 (ref.)
aOR CI 95%		0.90 (0.76–1.06)	1 (ref.)
**Haemorrhoids** ^**a**^			
No	1603	847 (56.4)	756 (50.8)
Yes	1387	656 (43.6)	731 (49.2)
cOR CI 95%		**0**.**80** (**0**.**69**–**0**.**93**)	1 (ref.)
aOR CI 95%		**0**.**80** (**0**.**68**–**0**.**94**)	1 (ref.)
**Infected wound** ^**b**^			
No	2779	1364 (90.8)	1415 (95.2)
Yes	211	139 (9.2)	72 (4.8)
cOR CI 95%		**2**.**00** (**1**.**49**–**2**.**69**)	1 (ref.)
aOR CI 95%		1.08 (0.77–1.50)	1 (ref.)
**Perineal painª**			
No	1729	821 (54.6)	908 (61.1)
Yes	1261	682 (45.4)	579 (38.9)
cOR CI 95%		**1**.**30** (**1**.**30**–**1**.**51**)	1 (ref.)
aOR CI 95%		**1**.**26** (**1**.**05**–**1**.**51**)	1 (ref.)
**Headache** ^**c**^			
No	2313	1184 (78.8)	1129 (75.9)
Yes	677	319 (21.2)	358 (24.1)
cOR CI 95%		**0**.**85** (**0**.**72**–**1**.**01**)	1 (ref.)
aOR CI 95%		**0**.**75** (**0**.**61**–**0**.**91**)	1 (ref.)
**Breast pain** ^**d**^			
No	1728	811 (54.0)	917 (61.7)
Yes	677	692 (46.0)	570 (38.3)
cOR CI 95%		**1**.**37** (**1**.**19**–**1**.**59**)	1 (ref.)
aOR CI 95%		**1**.**34** (**1**.**14**–**1**.**58**)	1 (ref.)
**Back pain** ^**e**^			
No	1676	838 (55.8)	838 (56.4)
Yes	1314	665 (44.2)	649 (43.6)
cOR CI 95%		1.03 (0.89–1.18)	1 (ref.)
aOR CI 95%		0.89 (0.76–1.05)	1 (ref.)
**Burning during urination** ^**f**^			
No	2430	1195 (79.5)	1235 (83.1)
Yes	560	308 (20.5)	252 (16.9)
cOR CI 95%		**1**.**26** (**1**.**05**–**1**.**52**)	1 (ref.)
aOR CI 95%		**1**.**31** (**1**.**06**–**1**.**61**)	1 (ref.)
**Urinary Incontinence** ^**g**^			
No	2007	1003 (66.7)	1004 (67.5)
Yes	983	500 (33.3)	483 (32.5)
cOR CI 95%		1.04 (0.89–1.21)	1 (ref.)
aOR CI 95%		1.03 (0.86–1.23)	1 (ref.)
**Faecal incontinence** ^**g**^			
No	2884	1406 (93.5)	1438 (96.7)
Yes	146	97 (6.5)	49 (3.3)
cOR CI 95%		**2**.**03** (**1**.**43**–**2**.**88**)	1 (ref.)
aOR CI 95%		**1**.**60** (**1**.**07**–**2**.**38**)	1 (ref.)
**Fatigue** ^**h**^			
No	459	214 (14.2)	245 (16.5)
Yes	2531	1289 (85.8)	1242 (83.5)
cOR CI 95%		1.19 (0.97–1.45)	1 (ref.)
aOR CI 95%		0.97 (0.78–1.22)	1 (ref.)
**Sadness** ^**i**^			
No	1648	700 (46.6)	948 (63.8)
Yes	1342	803 (53.4)	539 (36.2)
cOR CI 95%		**2**.**02** (**1**.**74**–**2**.**34**)	1 (ref.)
aOR CI 95%		**1**.**60** (**1**.**35**–**1**.**89**)	1 (ref.)
**Anxiety** ^**i**^			
No	2049	944 (62.8)	1105 (74.3)
Yes	941	559 (37.2)	382 (25.7)
cOR CI 95%		**1**.**71** (**1**.**47**–**2**.**00**)	1 (ref.)
aOR CI 95%		**1**.**34** (**1**.**12**–**1**.**61**)	1 (ref.)
**Depression** ^**i**^			
No	2485	1170 (77.8)	1315 (88.4)
Yes	505	333 (22.2)	172 (11.6)
cOR CI 95%		**2**.**18** (**1**.**78**–**2**.**66**)	1 (ref.)
aOR CI 95%		**1**.**65** (**1**.**31**–**2**.**06**)	1 (ref.)
**Sexual problems** ^**j**^			
No	1815	801 (53.3)	1014 (68.2)
Yes	1175	702 (46.7)	473 (31.8)
cOR CI 95%		**1**.**87** (**1**.**62**–**2**.**18**)	1 (ref.)
aOR CI 95%		**1**.**46** (**1**.**23**–**1**.**73**)	1 (ref.)
**Relationship problems** ^**j**^			
No	2092	980 (65.2)	1112 (74.8)
Yes	898	523 (34.8)	375 (25.2)
cOR CI 95%		**1**.**58** (**1**.**35**–**1**.**85**)	1 (ref.)
aOR CI 95%		**1**.**43** (**1**.**20**–**1**.**71**)	1 (ref.)
**Lactation problems** ^**i**^			
No	1897	777 (51.7)	1120 (75.3)
Yes	1093	726 (48.3)	367 (24.7)
cOR CI 95%		**2**.**85** (**2**.**44**–**3**.**33**)	1 (ref.)
aOR CI 95%		**2**.**46** (**2**.**05**–**2**.**94**)	1 (ref.)

cOR: Crude Odds Ratio Crude; aOR: Adjusted Odds Ratio.

^a^Adjusted by maternal age, academic level, maternal training, multiple birth, type of delivery, episiotomy and severe tearing.

^b^Adjusted by maternal age, academic level, maternal training, type of delivery, episiotomy, severe tearing, complications during pregnancy and type of lactation.

^c^Adjusted by maternal age, academic level, maternal training, type of delivery, multiple birth, epidural use/spinal anesthesia, complications during pregnancy and type of lactation.

^d^Adjusted by maternal age, academic level, maternal training, type of delivery, multiple delivery, complications during pregnancy and type of lactation.

^e^Adjusted by maternal age, academic level, maternal training, type of delivery, multiple delivery, complications during pregnancy, epidural use/spinal anesthesia, gestational age and type of lactation.

^f^Adjusted by maternal age, academic level, maternal training, type of delivery, complications during pregnancy, episiotomy, severe tearing, epidural use/spinal anesthesia, gestational age and type of lactation.

^g^Adjusted by maternal age, academic level, maternal training, type of delivery, multiple delivery, complications during pregnancy, epidural use/spinal anesthesia y gestational age.

^h^Adjusted by maternal age, academic level, maternal training, type of delivery, episiotomy, severe tearing, complications during pregnancy, type of lactation, multiple delivery and newborn admittance.

^i^Adjusted by maternal age, academic level, maternal training, type of delivery, episiotomy, severe tearing, complications during pregnancy, induced birth, epidural use/spinal anesthesia, type of lactation, nationality, gestational age, skin-to-skin, multiple delivery and newborn admittance.

^j^Adjusted by maternal age, academic level, maternal training, type of delivery, episiotomy, severe tearing, complications during pregnancy, induced birth, epidural use/spinal anesthesia, type of lactation, nationality, gestational age, multiple delivery and newborn admittance.

## Discussion

Our results identified an association between parity and various problems and discomforts that women suffer during the puerperium. Primiparous women, as compared to multiparous, are less likely to have haemorrhoids and headaches. However, they have an increased risk of having lactation complications, sexual problems, problems in the habitual dynamics of the couple, faecal incontinence, burning during urination, perineal pain, breast pain, depression, anxiety, and sadness symptoms.

Among the factors associated with parity, from our results one can get that age ≥35 years was associated with being multiparous. This may be due to the fact that being older increases the chances of having had a previous pregnancy. Likewise, women who perform more skin-to-skin contact with their newborn are also multiparous compared to primiparous. In this same line, in a Swedish study on 64 newborns, 54.7% of the multiparous kept early skin-to-skin contact compared to 45.3% of the primiparous^14^. Other clinical variables and practices such as artificial feeding at discharge, the realization of an episiotomy, which produces a severe perineal tearing of III or IV degree during the delivery, the use of epidural, having a dystocia, the onset of a delivery in a non-spontaneous way, attending childbirth classes during pregnancy, and having a multiple pregnancy were positively associated with primiparity, in line with what is established in most scientific literature^9,13,15–20^.

Being multiparous is a risk factor for having haemorrhoids during the puerperium, in the line of what Jong-Hyun *et al*. detected in a study in Korea^21^ and Poskus *et al*. in a study with 280 women a month after childbirth conducted in Lithuania^22^. Also, multiparity was associated with greater headaches in the puerperium, as Sharff *et al*. already stated^23^.

Women who have their first birth presented more frequent perineal pain in the puerperium, also in line with the results of other authors^22^. Also, women who were in their first birth showed a greater presence of depressive, anxious and sad feelings, and they also rekindled the delivery upsettingly. All these feelings can be due to cortisol levels, very mood-related. Gillespie *et al*. detected an interaction between parity and cortisol levels^24^. However, Hartmann *et al*.^25^, in the opposite direction of our results, identified the association between multiparity and postpartum depression.

On the other hand, the primiparous showed more sexual problems in the puerperium than the multiparous. In this sense, the results of the study by Yee *et al*.^26^, carried out on 160 postpartum women, show an earlier retake of sexual relations and with greater satisfaction in the multiparous, in the same line with our results.

Discomfort and burning when urinating, breast pain and faecal incontinence were more frequently in primiparous women.

Prinds *et al*.^27^, in a survey on 499 women in Denmark, concluded that the first child birth had forged stronger ties between couples, as opposed to the results of our study. Although the results of Mchale & Huston^28^ show that the transition to paternity affects fellowship and marital role patterns, no evidence was found to support the idea that paternity is associated with a decrease in mutual assessments (love) or marriage assessment (marital satisfaction).

Regarding lactation, primiparous women had a higher risk of having problems than multiparous, perhaps due to the experience acquired with previous children. Our results go in line with other authors^9,29–31^.

In the case of a non-response selection bias, it has had no influence on the results. Women’s response to the participation has been preponderant. Only 29 refused to participate (13 multiparous, 0.82% of all multiparous, and 16 primiparous, 0.92% of the primiparous participants) and nothing suggests that those who did not respond would have done so differently from those that did. Likewise, both in the multiparous and primiparous groups, the rejection to participate was similar: 0.82% vs 0.92%. The existence of an information bias is unlikely: the data collected, as well as the way in which the possible responses were posed, do not require a high level of education. The questions were presented in a basic and simple, affordable and comprehensible way for any educational level. It is not completely possible to reject a recall bias, although the information was collected in a short interval of time. Therefore, if there was an influence on the results, we believe it would have been minimal. Women especially remember the information on their birth process, which is generally highly valued and deserves special attention on their part. It is not possible to ignore a residual confusion bias, even if the influence on the results would have been minimal, since the adjustment of each variable has been carried out in an individualised and specific way through those variables that have possibly influenced this specific one.

Among the strengths of the study, we must highlight that the sample size is large, with women from different geographical areas, so it includes all the possible sensitivities of the reference population.

## Conclusion

In conclusion, parity is associated with the type of discomfort and problems that women have in the puerperium. Primiparous women show more symptoms related to mental health such as anxiety, depression and sadness, as well as couple dynamics and sexuality problems, among others. The multiparous women presented a greater frequency of haemorrhoids and headaches in the postpartum period.

## Methods

A cross-sectional study was conducted with women who gave birth in Spain in 2017 (both in public health system centres and private centres). Births with antepartum stillbirths and women under 18 years of age were excluded.

For the sample size estimation, the criterion of maximum modelling was considered^32^. This implied including 10 events (complications) for each independent variable that was to be incorporated in the multivariate analysis. Considering faecal incontinence^33^ as the less frequent complication (4%), a minimum of 2500 women were required to incorporate a minimum of 10 independent variables (100 × 100/4 = 2500).

### Sources of information

For the data collection, a self-elaborated online questionnaire of 35 items (3 open questions and 32 closed questions) was given to the women 6 weeks after the delivery. Data was collected on sociodemographic and clinical characteristics, obstetric outcomes, newborns data and complications, discomfort and needs of postpartum women. The questionnaire was piloted by the researchers and midwives who would recruit the participants for the study. It was piloted with women from different Spanish geographical areas, with different levels of studies and ages. The Associations of Spanish Midwives Federation (FAME, for its acronym in Spanish), as well as their member associations, were involved in the dissemination of the project and in the recruitment of participants. These involved their midwives in the dissemination of project and in the recruitment of participants. These midwives helped in the recruitment of women and were also trained to explain and support women in completing the questionnaire if necessary. Once the study subjects were selected and they had agreed to participate, they were given the instructions to complete the questionnaire (self-administered), which they filled according to their availability. There was a telephone number and a chat destined to answer all the possible questions these women may have had in completing the questionnaire.

#### The following variables were collected

The main outcomes were women symptoms of: constipation problems, presence of haemorrhoids, wound infection (need of professional cures after discharge from hospital and/or consumption of antibiotics, perineal pain, headache, breast pain, back pain, pains or burning sensation during urination, faecal incontinence (inability to control bowel movements), urinary incontinence (involuntary urine loss), tiredness, sadness, anxiety (nervousness and/or restlessness), depression (depressive mood), problems related to maintaining sexual intercourse, problems in couple’s relationship dynamics after birth, and problems related to lactation. All the dependent variables were dichotomous (yes/no).

### Statistical analysis employed

The main independent variable was parity (Primiparous: a woman in her first birth; Multiparous: a woman in her second or further birth).

The variables considered for the confounding control were of demographic (maternal age, academic level, maternal training, nationality) and clinical type (type of delivery: type of birth: being eutocic, instrumental, or performing a caesarean), multiple birth (childbirth with more than one newborn), complications during pregnancy (appearance of haemorrhages, fever, need for surgical intervention after delivery, among others), induced birth (labour that does not start spontaneously, medicines are used to start it), epidural use/spinal anesthesia (analgesia that is administered through the epidural space), episiotomy (surgical incision that is made in the perineum to facilitate the expulsion of the foetus), severe perineal tearing (spontaneous lesion of the perineal that occurs during the expulsion of the foetus, tears of III or IV degree that affect the integrity of the anal sphincter), gestational age (week of pregnancy at which labour occurs), skin-to-skin (recommended clinical practice consisting of establishing direct contact between the newborn and the mother: skin to skin), newborn admittance (need for hospital admission of the newborn), and type of lactation (type of lactation with which the newborn is fed: exclusively breastfeeding, mixed lactation, or exclusively with artificial formulas), using in each case the specific variables that could potentially act as confusing for each specific result, that is, individualising the adjustment variables for each result.

Firstly, a descriptive analysis was carried out with absolute and relative frequencies. Then, a bivariate analysis was carried out between parity and the main manifestations/complications/discomforts that women showed through binary logistic regression. Later, a multivariate analysis was carried out through logistic regression, using SPSS forward and backward selection. The aim of this analysis was to determine the net effect of parity on each postpartum problem/type of discomfort. For each analysis, the potentially confounding variables were included, following clinical criteria.

Crude odds ratios (cOR) and adjusted odds ratios (aOR) and their confidence intervals (CI) were calculated at 95%.

A p < 0.05 was considered as significant. All analyses were carried out with the SPSS v24.0 statistical package.

### Ethics approval

This study was approved by the Ethical Committee on Clinical Research (CEIC, for its Spanish acronym) of the La Mancha-Centro Centre with ethical code 69-C. Before starting the questionnaire, the participating women read a fact sheet about the study, its objectives, etc., and marked a box by which they showed their consent to participate in it, i.e., they signed an online informed consent (ticking the option if they wanted to participate or not doing so when refusing to take part in the study). The protocols established to carry out this type of research were followed with the purpose of publication/disclosure to the scientific community. The study was conducted according to the guidelines set in the Declaration of Helsinki and all procedures involving human subjects were approved by the Ethics Committee. All women involved in this study filled out an informed consent and data treatment forms to enter the study, in accordance with the ethical standards of the Ethics Committee.

## Data Availability

The datasets generated during and/or analysed during the current study are available from the corresponding author on reasonable request.
